# Metabolism of *N*-Acylated-Dopamine

**DOI:** 10.1371/journal.pone.0085259

**Published:** 2014-01-22

**Authors:** Dominika Zajac, Grzegorz Spolnik, Piotr Roszkowski, Witold Danikiewicz, Zbigniew Czarnocki, Mieczyslaw Pokorski

**Affiliations:** 1 Department of Respiratory Research, Medical Research Center, Polish Academy of Sciences, Warsaw, Poland; 2 Mass Spectrometry – Group One, Institute of Organic Chemistry, Polish Academy of Sciences, Warsaw, Poland; 3 Laboratory of Natural Products Chemistry, Faculty of Chemistry, Warsaw University, Warsaw, Poland; University of Iowa, United States of America

## Abstract

*N*-oleoyl-dopamine (OLDA) is a novel lipid derivative of dopamine. Its biological action includes the interaction with dopamine and the transient receptor potential vanilloid (TRPV1) receptors. It seems to be synthesized in a dopamine-like manner, but there has been no information on its degradation. The aim of the study was, therefore, to determine whether OLDA metabolism proceeds the way dopamine proper does. We addressed the issue by examining the occurrence of *O*-methylation of exogenously supplemented OLDA *via* catechol-*O*-methyltransferase (COMT) under *in vitro*, *ex vivo*, and *in vivo* conditions using rat brain tissue. The results show that OLDA was methylated by COMT in all conditions studied, yielding the *O*-methylated derivative. The methylation was reversed by tolcapone, a potent COMT inhibitor, in a dose-dependent manner. We conclude that OLDA enters the metabolic pathway of dopamine. Methylation of OLDA may enhance its bioactive properties, such as the ability to interact with TRPV1 receptors.

## Introduction

The subject of the present study was *N*-oleoyl-dopamine (OLDA), N-[2-(3,4-dihydroxyphenyl) ethyl]-9Z-octadecenamide, a novel lipid derivative of dopamine [1]–[3]. The compound is synthesized by direct *N*-acylation of dopamine (DA) by oleic acid followed by a standard sequence of reactions leading to the formation of a DA moiety [4]. Although OLDA is present endogenously in mammalian brain [4], neither its metabolism nor the biological role has been well explored. Exogenously applied OLDA is taken up by both central and peripheral neural tissues [5], [6]. OLDA seems to be more stable *in vitro* than dopamine proper is [7], [8] and some of OLDA major bioactivities noted in the experimental studies, such as enhancement of locomotor activity [7] or lowering the reserpine-induced muscle rigidity [9], are mediated *via* the dopamine pathway. OLDA also is a ligand for the transient receptor potential vanilloid V1 (TRPV1) receptors [4], [10], [11]. These receptors seem to favor the compounds that have the 3-methoxy-4-hydroxybenzyl (homovanillyl) and the 3,4-dihydroxybenzyl (catechol) groups [12], [13], which they are affiliated with. The latter group is present in OLDA and in its methylated metabolite – 3-methoxy-*N*-oleoyl-dopamine (*O*-Me-OLDA). Thus, OLDA has a preferable structure regarding the affiliation with TRPV1 receptors, and indeed is a proven TRPV1 agonist [14]. The question arises of whether *O*-methylation of OLDA could occur in the biological setting and whether it is catechol-*O*-methyltransferase (COMT)-dependent. We addressed this question by examining the ability of exogenously supplemented OLDA to undergo the methylation process in *in vitro*, *ex vivo*, and *in vivo* conditions. The study gave a positive answer to the question posed, which gives supportive evidence for a dopamine-like metabolic pathway of OLDA and raises the possibility that pre-methylation of OLDA facilitates its affinity to TRPV1 receptors.

## Materials and Methods

### 1. Ethics statement

The study was approved by the IV Warsaw Local Ethics Committee for Animal Experiments (Permit Number: 81/2009) and it was performed in accordance with the Guiding Principles for the Care and Use of Laboratory Animals of the American Physiological Society [15].

### 2. Outline of protocol and animal tissue

This basically biochemical study consisted of the *in vitro*, *ex vivo*, and *in vivo* experiments. Firstly, we examined whether OLDA would undergo the *O*-methylation *in vitro* catalyzed by a commercially available COMT. Secondly, we determined whether *O*-methylation of OLDA would be possible in an *ex vivo* tissue in the presence of endogenous COMT, according to the method of Brannan et al. (16]. Finally, we sought to determine the presence of *O*-Me-OLDA in brain tissue after intraarterial injection of OLDA *in vivo*. The biological material used for the assays was the rat brain tissue obtained from a total of 17 adult male Wistar rats. The animals were surgically anesthetized with intraperitoneal injection of urethane and α-chloralose (700 and 120 mg/kg, respectively). The monitoring of the state of the pupil was used as an indication of the level of anesthesia. Supplemental doses of the anesthetics, 10% of the original dose, were given when a painful stimulus, such as the piercing of a hind paw, caused a rapid reaction of the pupil. At the end of the experimental procedures, the animals were sacrificed by perfusion through the left heart with icy cold 0.9% NaCl, the brains were rapidly removed, and the homogenates were prepared as below outlined. To prove that the methylation is COMT-dependent, we performed additional experiments with and without inhibition of COMT by tolcapone. All reagents used in the study were of analytical grade and were acquired from Sigma-Aldrich (St. Louis, MO), except for tolcapone that was obtained from KeyOrganics (Camelford, UK).

### 3. Syntheses of OLDA and *O*-Me-OLDA

Syntheses of OLDA and *O*-Me-OLDA were performed according to the method of Czarnocki et al. [2]. Briefly, 0.43 g (2.27 mmol) of dopamine hydrochloride, 0.64 g (2.27 mmol) of oleic acid, 1.00 g (2.27 mmol) of benzotriazol-1-yloxytris(dimethylamino)-phosphoniumhexafluorophosphate (BOP) and 20 ml of tetrahydrofuran (THF) were introduced into a flask. The reaction mixture was cooled to 0–5°C and 0.95 ml (6.81 mmol) of triethylamine in 4 ml THF were added dropwise within 15 min, and the reaction mixture was continuously stirred for 15 h at room temperature. After evaporating the solvent, 100 ml of diethyl ether were added and the ether phase was washed three times with 30 ml of a 4% HCl solution, twice with 30 ml of a saturated NaHCO_3_ solution and twice with a saturated NaCl solution. After drying over anhydrous MgSO_4_ and removal of the solvent, the crude product was purified from diethyl ether/hexane by crystallization, which gave 0.90 g (92% yield) of the amide as a white solid. In the synthesis of *O*-Me-OLDA, dopamine hydrochloride was replaced by 0.46 g (2.27 mmol) of 3-*O*-methyl-dopamine hydrochloride.

### 4. *In vitro* OLDA *O*-methylation - commercial COMT added

The reaction was performed in a reaction buffer (RB) containing phosphate buffered saline (PBS), pH 8.0, and 10 mM Mg^2+^. Briefly, 0.5 mg of COMT, derived from porcine liver, with the activity of 150 U/mg protein (basal activity 1.107 µmol substrate per hour) in 1% bovine serum albumin (BSA), 0.87 mg (1.11 µmol) of S-adenosyl-L-methionine (SAM) and 0.46 mg (1.11 µmol) of OLDA in a drop of Tween80 were dissolved in a volume of 0.1 ml each, mixed, and then the RB was added to a total volume of 0.5 ml. The reaction mixture and the control mixture (in which COMT was replaced by RB) were kept for 75 min at 37°C. After the reaction time, both solutions were centrifuged for 10 min at 1200× *g*. The supernatant was extracted 4×0.25 ml CHCl_3_ giving the organic and aqueous phases. In the organic phase (containing CHCl_3_ as solvent) the hydrophobic compounds OLDA and *O*-Me-OLDA, the supposed products of the reaction, were expected. In contrast, in the aqueous phase, all ions and hydrophilic compounds like SAM and its demethylation product, S-adenosyl-L-homocysteine (SAH), were to be found. The organic phase was dried under N_2_, the aqueous phase was dried in a vacuum evaporator, and both phases were analyzed spectrophotometrically. An additional control consisting of a reaction mixture, with the same content of ingredients, analyzed 1 min after the commencement of the reaction, showed that the UV/VIS spectra were not different from those analyzed in the control solution above outlined. Therefore, the presence of COMT had no influence on the spectra.

### 5. *Ex vivo* OLDA *O*-methylation - endogenous COMT in brain tissue

COMT preparations were obtained as described by Brannan et al. [16]. Briefly, three fresh rat brains were homogenized in 4 v/m of 0.15 M KCl. The homogenate was centrifuged at 15000× *g* for 40 min. Then, the pellet was discarded and the supernatant was used as the enzyme preparation. Five milligrams of OLDA were dissolved in one drop of Tween80; then 250 µl of the supernatant, 2 mg of SAM, 50 µl of 5 mM MgCl_2_, and 1.7 ml of calcium-free PBS were admixed. In the control solutions, OLDA was omitted. After 1 h of incubation at 37°C, the reaction was stopped by the addition of 0.4 ml of 8% trichloroacetic acid and the proteins were precipitated by centrifugation at 2000× *g* for 5 min at 4°C. Then, the lipophilic compounds were extracted 4 times with 1 ml of chloroform, both phases dried and analyzed by HPLC-MS.

### 6. *In vivo* OLDA *O*-methylation

Three anesthetized rats were used in this part of the study. A canula was introduced upstream into a carotid artery to inject OLDA in a dose of 40 mg/kg dissolved in 0.3 ml of dimethyl sulfoxide (DMSO). One hour later, the animals were sacrificed as above outlined. Then, brains were enucleated from the skull, weighed, homogenized in 20 v/m of chloroform∶methanol 2∶1 v/v and left overnight at 4°C. Later, the mixture was dried under N_2_ to a volume of 3 ml and lipids were extracted 3 times with chloroform and water (1∶1 v/v, 4 ml). The organic, lipid-containing fraction was dried under nitrogen and stored at −80°C until HPLC-MS analysis. Another three anesthetized control rats received 0.3 ml of DMSO alone and otherwise underwent the same experimental procedure.

### 7. COMT inhibition by tolcapone

In the *in vitro* part of the study, 1 µM of tolcapone was added to the reaction mixture 20 min before OLDA and then the reaction was allowed to proceed as outlined in section 2.3. In the *ex vivo* part of the study, brains extracted from two rats were used. After homogenization and centrifugation, as described in section 2.4, 250 µl of the supernatant, containing endogenous COMT, 2 mg of SAM, 50 µl of 5 mM MgCl_2_, and 1.7 ml of calcium-free PBS were mixed with tolcapone at a final concentration of 0.1 and 1 µM. The mixture was incubated 20 min at 37°C. Then, 5 mg of OLDA dissolved in one drop of Tween80 were added to the reaction mixture and incubated for 1 h at 37°C. The reaction proceeded as outlined in section 2.4. In the control solutions, OLDA was omitted. Each assay was performed in triplicate.

Six anesthetized animals were used for the *in vivo* tolcapone part of the study. The inhibitor was injected at a dose of 15 or 30 mg/kg, i.p., in three animals each. Two hours later, the animals were prepared surgically and 40 mg/kg OLDA was injected into the carotid artery. The remaining procedure was as above described in the *in vivo O*-methylation (section 2.5).

### 8. Analytical methods

#### 8.1. UV/VIS spectrophotometry

Organic phases of the samples obtained were dissolved in 0.3 ml of CHCl_3_, whereas the aqueous phases were suspended in 0.3 ml of methanol. All samples were analyzed spectrophotometrically using a Cintra 10e UV/VIS Spectrophotometer equipped with Spectral 1.70 (GBC Scientific Equipment Pty Ltd., Victoria, Australia) in a range of 190–1000 nm, with 0.427 nm steps. Spectrophotometric measurements were performed immediately after the extraction procedures.

#### 8.2. HPLC-MS analysis

Samples for the HPLC-MS experiments were diluted in 1 ml of dichloromethane (DCM) and 20 µl of the solution was mixed with 1 ml of methanol. A standard mix of OLDA or *O*-Me-OLDA was prepared by diluting 1 mg of either in 1 ml of DCM, further diluted 100 times with methanol. The HPLC-MS experiments were carried out using a high-performance liquid chromatographic prominence LC-20 (Shimadzu, Kyoto, Japan) coupled with a tandem mass spectrometer 4000 Q TRAP (Applied Biosystems, Carlsbad, CA). The mass spectrometer was equipped with an electrospray ion source (Turbo Ion Spray) and a triple quadrupole/linear ion trap mass analyzer.

HPLC separation was performed using a 4.6×50 mm Luna C8 (5 µm) column (Phenomenex, Torrance, CA). A linear multistep gradient was used in all measurements. The elution started from 20% water in methanol and reached 70% water in methanol at 12 min. This composition of the eluent was kept for 5 min and during the following one minute, the amount of water in methanol was increased to 80%; the level being maintained for further 2 min. The flow rate was 2.0 ml/min.

Multiple reaction monitoring (MRM) experiments were performed in the negative ion mode. Zero air was used as a nebulizer and nitrogen was used as a curtain gas. The tip voltage was kept at −4500 V and the declustering potential was set at 60 V. For all MRM experiments, the collision energy was set to 30 eV and the dwell time to 200 ms.

### 9. Data elaboration

Data are given as means ±SE counts of ions of fragmentation of OLDA and *O*-Me-OLDA. The number of fragmentation ions for OLDA corresponds closely to the residual/remaining OLDA. On the basis of these values, the percentage of conversion of OLDA to *O*-Me-OLDA was calculated. Differences in the *in vivo O*-methylation yield of OLDA obtained in the presence of endogenously acting COMT and COMT inhibited by the two doubling doses of tolcapone were assessed with the Kruskal-Wallis test. Statistical significance of differences was set at P<0.05.

## Results

### 1. *In vitro* OLDA *O*-methylation - commercial COMT added

**Figure 1:**
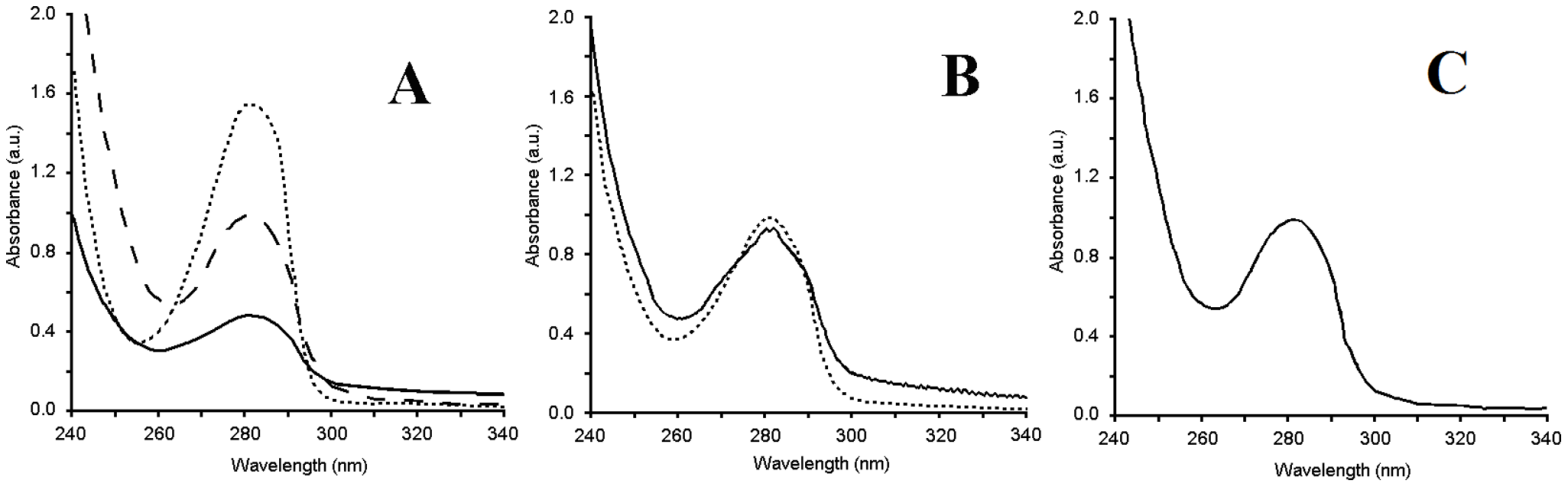
Methylation *in vitro* using commercially available COMT presented by the spectra of organic phases. (A) OLDA with COMT (continuous line); OLDA without COMT (dashed line); and *O*-Me-OLDA alone, a presumed reaction product, (dotted line). (B) OLDA with COMT (continuous line) and 1∶1 artificial mixture of OLDA and *O*-Me-OLDA (dotted line). The identity of the two spectra in Panel B points to the presence of OLDA and *O*-Me-OLDA in the reaction mixture. (C) Spectrum of the organic phase of the control mixture with no COMT added, incubated for the same length of time as was the full reaction with COMT.

Spectra of the organic phases had the maxima at 278–282 nm in both control mixture (OLDA with no COMT; Figure 1A – dashed line) and reaction mixture (OLDA with COMT; Figure 1A – continuous line). The spectrum of *O*-Me-OLDA alone, a presumed reaction product, is depicted by the dotted line in Figure 1A. Localization of the minimum changed from 262 nm in the control mixture to 260 nm in the reaction mixture. The minimum of the latter matched neither that of OLDA (substrate, λ_min_ = 262 nm) nor *O*-Me-OLDA (product, λ_min_ = 255 nm) (Fig. 1A), but was identical with the minimum of an artificially produced 1∶1 mixture of OLDA and *O*-Me-OLDA (Fig. 1B), which points to the occurrence of a reaction of *O*-methylation of OLDA *via* COMT, yielding both OLDA and *O*-Me-OLDA in the assay. Additionally, the aqueous phases were compared and no change in the spectral maxima (λ_max_ = 259 nm) was found. However, the minimum changed from 234 nm in the control to 237 nm in the reaction mixture; these minima were characteristic of SAM and SAH, respectively (data not shown).

### 2. *Ex vivo* OLDA *O*-methylation - endogenous COMT in brain tissue

The organic phase was analyzed using both UV/VIS and HPLC-MS. The UV/VIS spectra showed a maximum at 278–282 nm and a minimum at 260 nm and were identical with the spectrum of a 1∶1 mixture of OLDA and *O*-Me-OLDA. All these spectra corresponded closely to those in *in vitro O*-methylation, the graphic data are therefore not shown.

**Figure 2:**
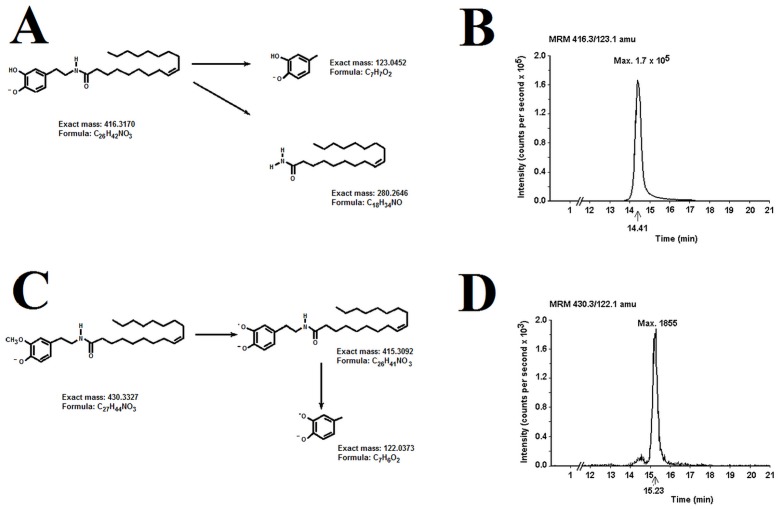
*O*-methylation of OLDA *in vivo* presented in the HPLC-MS spectra of brain extracts after intrarterial injection of OLDA. (A) Defragmentation of OLDA. (B) Chromatographic HPLC-MS peak typical for OLDA. The monitored ionic pair of OLDA of 416/123 *(m/z)* gave a retention time of 14.4. (C) Defragmentation of *O*-Me-OLDA. (D) Chromatographic HPLC-MS peak typical for *O*-Me-OLDA. The ionic pair of *O*-Me-OLDA of 430/122 *(m/z)* gave a retention time of 15.2. The signal at 430/122 indicates the presence of *O*-Me-OLDA in the sample (see text for details).

The organic phases obtained in the *ex vivo* experiments and also OLDA and *O*-Me-OLDA standards were analyzed using the HPLC-MS with multiple reaction monitoring (MRM) in the negative ion mode. The retention times were 14.4 and 15.2 min for the OLDA and *O*-Me-OLDA standards, respectively. The OLDA standard decomposed at the *m/z* values from 416 to 280 and to 123 and *O*-Me-OLDA at the *m/z* values from 430 to 415 and to 122. The leading pairs, 416/123 for OLDA and 430/122 for *O*-Me-OLDA, were chosen for further analysis and detection in the quadrupole system. An equimolar mixture of OLDA and *O*-Me-OLDA gave comparable intensities of the peaks (1.7×10^4^ counts per sec for OLDA and 1.9×10^4^ counts per sec for *O*-Me-OLDA), enabling the calculation of an approximate semi-quantitative ratio of methylation. The fragmentation patterns of OLDA and *O*-Me-OLDA are shown in Fig. 2 (Panels A and C, respectively).

Fractions with the retention times of 14.3–14.6 and 15.1–15.6 min were taken for MS analysis. A fragmentation pattern of 430/122 (*m/z*) (typical for *O*-Me-OLDA) was observed at a retention time of 15.5–15.6 min, whereas a pattern of 416/123 (*m/z*), typical for OLDA, was observed at 14.1–14.2 min in different experiments. These shifts in retention times were due to the presence of other compounds in the matrix. Likewise, the intensity of the peaks varied from 575 to 650 (mean 613±37) counts per sec for *O*-Me-OLDA and from 2.0×10^5^ to 2.1×10^5^ (mean 2.05±0.05×10^5^) counts per sec for OLDA. The ratio of methylated to total OLDA used in the reaction mixture varied from 0.028 to 0.030 (mean 0.029±0.001), giving a methylation yield of about 3%.

### 3. *In vivo* OLDA *O*-methylation

In brain homogenates obtained from the *in vivo* experiments, where OLDA was given intra-arterially, chromatographic HPLC-MS peaks with a retention time of 14.3–14.5 and 15.1–15.2 min were observed. Like in the *ex vivo O*-methylation above outlined, shifts in the retention times toward standards were due to the presence of other compounds in the extraction mixture. The 416/123 (*m/z*) ion pair (typical for OLDA) at 14.3–14.5 min had an intensity of 7.4×10^4^ to 1.7×10^5^ (mean 1.1±0.3×10^5^) counts per sec, and the 430/122 (*m/z*) ion pair (typical for *O*-Me-OLDA) at 15.1–15.2 min had an intensity of 515 to 1855 (mean 1165±474) counts per sec, depending on the experiment. In this experimental part, the ratio of methylated to total OLDA varied from 0.063 to 0.104 (mean 0.088±0.016), giving a methylation yield of 6.3 to 10.4 (mean 8.8±1.6)%. The relevant HPLC/MS/MS chromatograms typical for OLDA and *O*-Me-OLDA are exemplified in Fig. 2 (Panels B and D, respectively).

### 4. COMT inhibition by tolcapone

To demonstrate that *O*-methylation involves the COMT pathway, we performed experiments in which COMT was inhibited by tolcapone. In the *in vitro* part of the study, the spectra of control (no COMT) and reaction (with COMT and 1 µM tolcapone) mixtures were identical, with the maxima and minima at 278–282 nm and 262 nm, respectively, which were the same as those noted for OLDA. In the *ex vivo* part, 1 and 0.1 µM concentrations of tolcapone were used. At either concentration no fragmentation ions of 430/122 (*m/z*) (typical for *O*-Me-OLDA) were found. The mean number of counts per sec at 416/123 (*m/z*) (typical for OLDA) varied from 3.7±0.1×10^5^ to 4.2±0.3×10^5^ for 0.1 and 1 µM tolcapone, respectively, p>0.05.

*In vivo*, two doubling doses of tolcapone of 15 and 30 mg/kg, i.p., were used. At the lower dose, the 416/123 (*m/z*) ion pair (typical for OLDA), had an intensity of 6435 to 12000 (mean 9245±1724) counts per sec, and the 430/122 (*m/z*) ion pair (typical for *O*-Me-OLDA) had an intensity of 20 to 45 (mean 30±9) counts per sec; giving a methylation yield of 0.238–0.373 (mean 0.3165±0.049)%. At the higher dose, the respective intensities were of 9.7×10^4^ to 1.4×10^5^ (mean 1.2×10^5^±0.2×10^5^) counts per sec and of 120 to 210 (mean 148±38) counts per sec; giving a further reduced methylation yield down to 0.109–0.139 (mean 0.122±0.010)%. The methylation yields obtained during *in vivo O*-methylation in the presence of endogenous COMT, with no tolcapone added (as outlined in Section 3.3), and after injections of two doses of tolcapone, were significantly different from one another (p<0.023).

## Discussion

The major finding of this study was that exogenously supplemented OLDA underwent COMT-catalyzed *O*-methylation. The methylation process was confirmed both *in vitro*, when commercial COMT was introduced into the assay, and *ex vivo*, when COMT native to the animal's brain tissue was the catalyst of the reaction. After systemic OLDA administration *in vivo*, *O*-Me-OLDA was positively identified in MS spectra obtained from the rat brain; 6.4–10.5% yield of *O*-methylation of OLDA was obtained. Pretreatment with the COMT inhibitor tolcapone drastically reduced this yield down to 0.11–0.14% in a dose-dependent manner, demonstrating the preponderance of the COMT pathway for the methylation reaction. The essential role of COMT in the metabolism of OLDA was strengthened by the apparent lack of *O*-Me-OLDA in the *in vitro* and *ex vivo* assays in which COMT was inhibited. The identification of *O*-Me-OLDA and its way of formation expands the understanding of OLDA biotransformation and its potential physiological role.

A question arises as to whether endogenous OLDA could be a substrate for COMT. Since exogenous OLDA undergoes *O*-methylation *in vivo* and is directly methylated by commercially available and *ex vivo* prepared COMT, it is a reasonable assumption that endogenous OLDA would also undergo this reaction. However, endogenous OLDA, present in picomolar concentrations in the brain [4], was below the detection limit in the present experiment, which made it impossible to verify whether it could be a substrate for COMT. Chu et al. (4] have identified OLDA in the bovine brain as a compound having an MS pattern identical to that of artificially produced OLDA; however, its minute concentration apparently escaped quantitative determination. On the other hand, Huang et al. [17] quantified the amount of *N*-arachidonoyl-dopamine (NADA) at about 7 pmol/g wet tissue in the striatum, which approximates 3 ng/g wet tissue. As OLDA is believed to be an endovanilloid, belonging to the family of *N*-acylated-dopamines, as does NADA, it seems a reasonable assumption that the endogenous concentration of OLDA is in the same range. With that assumption in mind, in the present study we used the concentrations of OLDA of 2.2 mM (1.1 µmol/0.5 ml) in *in vitro* part and 6 mM (12.0 µmol/2 ml) in *ex vivo* part, which were non-physiologically high. These concentrations were based on the findings of Pokorski et al. [5] that one micromole of OLDA (the approximate amount used *in vitro* in the present study) is the presumed quantity of the compound to be present in 1 g of rat brain tissue after systemic administration of 40 mg/kg OLDA at a penetration yield of 6%.

The only available data on *O*-methylation of lipid derivatives of dopamine are those of Huang et al. [17] concerning NADA. NADA was methylated *ex vivo* by the soluble form of COMT from the liver at about 2 pmol/min per mg protein, which seems to be low compared with the values for dopamine methylation - *V*max from 50 pmol/min per mg protein in skeletal muscles to as high as 14,690 pmol/min per mg protein in the liver [18]. A slower turnover of *N*-acylated dopamines might underlie a much sustained bioactivity of these compounds [19] compared with that of dopamine proper; assuming that the kinetics of exogenous and endogenous *N*-acylated dopamines are similar; which, however, remains to be shown.

The formation of *O*-Me-OLDA is congruous with the notion that OLDA enters the dopamine metabolic pathway. COMT and MAO are the two main enzymes engaged in dopamine metabolism. Metabolic reactions run in two parallel ways: COMT methylates DA to 3-methoxytyramine, which is later oxidized by MAO to homovanillic acid (HVA), or MAO acts first yielding the 3,4-dihydroxyphenylacetic acid (DOPAC), which is later methylated by COMT to HVA [20]. It is believed that COMT is the predominant metabolic enzyme for brain dopamine [21]. Therefore, OLDA, which penetrates into the brain and stays there as a stable integral compound, would not only have a DA-like functional effects, such as increased locomotion or respiratory inhibition [7], which are blocked by DA antagonists, but would also engage the DA metabolic pathway. There are other possible ways of OLDA decomposition, which, albeit not tackled in the present study, seem rather unlikely. One of those is hydrolysis of OLDA at the amide bond. However, OLDA's lack of interaction with the fatty acid amide hydrolase (FAAH) [4] and its stability *in vivo* up to 24 h without traces of hydrolysis or auto-oxidation [19] speak against this possibility. Likewise, oxidation of the catechol ring, which in case of DA, especially under pathological conditions, gives rise to possibly toxic dopamine quinones [22], is unlikely in case of OLDA in mammalian neural tissue in which, as opposed to invertebrates [23], *N*-acyl-dopamine derivatives have not been found as substrates for tyrosinase-catalysed dopamine quinone formation. Thus, the most probable metabolic pathway of OLDA, apart from the possible sulfation reported previously by Akimov et al. [24], is its *O*-methylation.

Pre-methylation of OLDA could also have to do with its affinity to TRPV1 receptors [4], [10]. TRPV1 receptor ligands are mostly characterized by the presence of a 3-*O*-methylated catechol ring [25]. Capsaicin analogs, with an identical to OLDA 8-carbon lipophylic chain, containing *N*-methyl-amide in the B-region, and 3,4-dihydroxybenzyl (catechol) or 3-methoxy-4-hydroxybenzyl (3-methoxy-catechol or homovanillyl) group in the A-region are highly active regarding the transmembrane calcium influx [12], [13].The homovanillyl group is believed preferable for the interaction with the receptor. The catechol ring of OLDA is sufficient for the interaction with TRPV1, but *O*-methylation of OLDA would promote the interaction of its homovanillyl structure with TRPV1 receptors [26]. There seem to be satisfied experimental conditions to support this reasoning. In the present study we demonstrate the *in vivo O*-methylation of OLDA and both COMT and SAM, the latter a donor of the methyl group, are intracellular compounds [18], as also is the TRPV1 ligand binding structure. Despite the desirable presence of a methyl group for TRPV1 activation, the issue of the superior efficacy of *O*-Me-OLDA remains unresolved. There is a report showing that NADA activates TRPV1 with the potency similar to that of capsaicin [17], whereas the study examining the effect of *O*-Me-OLDA alone reported its potency toward TRPV1 lower than that of capsaicin [14]. It ought to be emphasized, however, that although NADA and OLDA belong to the same family of *N*-acylated dopamines, both compounds have different bioproperties. NADA is hydrolyzed to dopamine and arachidonic acid by FAAH [17], whereas OLDA has, in contrast, no interaction with this enzyme [4]. NADA is a ligand of CB1 cannabinoid receptors [27] and OLDA has no major activity at this receptor system. NADA synthesis proceeds *via* condensation of arachidonic acid and dopamine [27], rather than *via* enzymatic conversion of *N*-oleoyl tyrosine to OLDA as it is suggested in case of OLDA [4], although either way of synthesis for either compound seems a viable, albeit not clearly confirmed, possibility. The resolution of an issue of superiority of *O*-Me-OLDA over OLDA regarding the interaction with TRPV1 receptors ought to be based on a direct comparison of both compounds' affinities, which requires alternative study designs.

In synopsis, although physiological role of endogenous OLDA remains unclear, we believe we have conclusively demonstrated that the compound, supplemented exogenously, enters the metabolic pathway typical of dopamine and undergoes *O*-methylation by COMT *in vivo*. The study lends support for the dopamine-like properties of OLDA and suggests the possible molecular mechanism of its facilitated affinity to the methyl group-seeking TRPV1 receptors. OLDA carries a potential to incorporate lipid signaling into the hydrophilic dopamine pathway; which makes the compound of interest in studying the otherwise fleeting biological effects of dopamine. OLDA holds promise as a substitute for DA in DA deficient pathological states, which makes it worthwhile to further explore the properties of OLDA in exogenously bioactive doses.
